# Investigation of factors influencing the separation of diastereomers of phosphorothioated oligonucleotides

**DOI:** 10.1007/s00216-019-01813-2

**Published:** 2019-04-24

**Authors:** Martin Enmark, Maria Rova, Jörgen Samuelsson, Eivor Örnskov, Fritz Schweikart, Torgny Fornstedt

**Affiliations:** 1 Department of Engineering and Chemical Sciences, Karlstad University, 651 88 Karlstad, Sweden; 2 Pharmacognosy, Department of Medicinal Chemistry, Biomedical Centre, Uppsala University, Box 574, 75123 Uppsala, Sweden; 3 Advanced Drug Delivery, Pharmaceutical Sciences, IMED Biotech Unit, AstraZeneca, 431 83 Gothenburg, Sweden

**Keywords:** IP-RPLC, Ion-pair, Oligonucleotide, Antisense, Phosphorothioate, Diastereomer

## Abstract

This study presents a systematic investigation of factors influencing the chromatographic separation of diastereomers of phosphorothioated pentameric oligonucleotides as model solutes. Separation was carried out under ion-pairing conditions using an XBridge C_18_ column. For oligonucleotides with a single sulfur substitution, the diastereomer selectivity was found to increase with decreasing carbon chain length of the tertiary alkylamine used as an ion-pair reagent. Using an ion-pair reagent with high selectivity for diastereomers, triethylammonium, it was found the selectivity increased with decreased ion-pair concentration and shallower gradient slope. Selectivity was also demonstrated to be dependent on the position of the modified linkage. Substitutions at the center of the pentamer resulted in higher diastereomer selectivity compared to substitutions at either end. For mono-substituted oligonucleotides, the retention order and stereo configuration were consistently found to be correlated, with *R*p followed by *S*p, regardless of which linkage was modified. The type of nucleobase greatly affects the observed selectivity. A pentamer of cytosine has about twice the diastereomer selectivity of that of thymine. When investigating the retention of various oligonucleotides eluted using tributylammonium as the ion-pairing reagent, no diastereomer selectivity could be observed. However, retention was found to be dependent on both the degree and position of sulfur substitution as well as on the nucleobase. When analyzing fractions collected in the front and tail of overloaded injections, a significant difference was found in the ratio between *R*p and *S*p diastereomers, indicating that the peak broadening observed when using tributylammonium could be explained by partial diastereomer separation.

**Electronic supplementary material:**

The online version of this article (10.1007/s00216-019-01813-2) contains supplementary material, which is available to authorized users.

## Introduction

Antisense oligonucleotides (ASOs) are a growing and important class of therapeutic DNA or RNA intended for altering the function of RNA [1, 2]. ASOs have sequences that bind with high specificity to the RNA target through complementary base pairing. Inhibition of the target RNA is achieved through different mechanisms depending on the design of the ASO and the properties of the cell, and may or may not include cleavage of the target RNA [1].

Impurities are generated by the synthesis and degradation pathways of oligonucleotides [3, 4]. These impurities include shortmers and longmers, as well as depurination and deamination products [5–7]. For separation and quantification of impurities, ion-pair reversed phase chromatography is currently the dominant separation mode but ion exchange chromatography, capillary electrophoresis, and hydrophilic interaction chromatography are also used [3, 8, 9]. When using ion-pair chromatography, the most common ion-pairing reagents are tertiary alkylamines such as triethylammonium acetate (TEtAA) and tributylammonium acetate (TBuAA) [3, 10]. More recently, triethylamine with addition of hexafluoro-2-propanol (HFIP) has been introduced as a way to maintain chromatographic performance while also improving the mass spectrometry sensitivity [10].

Unmodified RNA and DNA are sensitive to endogenous nucleases and are also rapidly excreted from the body, ruling out their therapeutic applicability. Several kinds of modifications have been tested to overcome those problems, one of the most widely used being the phosphorothioation of ASOs. Incorporating a sulfur in place of one of the non-bridging oxygens of the phosphate group will, however, produce diastereomers with potentially different pharmacological and physicochemical characteristics. Stereospecific differences have been reported in nuclease resistance, RNase H activation, affinity to target RNA, and other pharmacological properties [11–14], clearly justifying study of the separation of diastereomers of phosphorothioated oligonucleotides. In this study, the diastereomers are referred to as in the *R*p or *S*p configuration based on their chirality on the modified phosphate group. The configuration at the phosphor can be determined by stereospecific enzymatic digestion of the phosphorothioated oligonucleotides [15–17].

If all phosphate linkages in an *n*-long oligonucleotide are phosphorothioate (PS) modified, this will lead to 2^*n* − 1^ diastereomers. For a 20-nucleotide-long PS-ASO, this exceeds 500,000 diastereomers. Many chromatographic methods can probably partially differentiate between these, leading to increased peak broadening due to poor diastereomer separation [3, 15, 18].

Chromatographic work concerning the separation of diastereomers began with the important work of Stec et al., who fully separated diastereomers of di- to hexadecamers of various oligonucleotides with up to two PS modifications using ion-pair reversed phase liquid chromatography with TEtAA [15]. Several authors have since then studied the diastereomer separation of PS-modified oligonucleotides, mostly utilizing reversed phase chromatography where up to 16 diastereomers have been separated [16, 17, 19–21]. The smaller degree of diastereomer selectivity has been found using anion exchange chromatography [16, 22]. It has also been indicated that capillary electrophoretic separation gives partial diastereomer separation [23]; a somewhat better separation was achieved utilizing a polymeric pseudo-stationary phase [24]. The current consensus is that chromatographic techniques cannot resolve all diastereomers in fully PS-modified oligonucleotides of lengths > 15 [3], and in reality, likely much shorter than that. Even using two-dimensional liquid chromatography, it has not been reported theoretical peak capacities beyond 15,000 [25]. If the stereochemistry of oligonucleotides is shown to be a crucial aspect of their efficacy and safety, stereospecific synthesis routes will likely be the only reasonable way to attain stereo purity [14]. Consequently, parts of the chromatographic community and industry have focused their efforts on developing methods to suppress diastereomer selectivity while maintaining compatibility with mass spectrometric detection [3, 26, 27].

The aim of this study is to systematically investigate how the choice of tertiary ion-pairing alkylamine, ion-pair concentration, and modifier gradient slope influence the diastereomer selectivity of short PS-modified oligonucleotides. As model compounds, PS pentamers of dT, dA, dC, and dG will be used; dT oligonucleotides are used to investigate how the degree and position of the sulfur substitution affect diastereomer separation when using a highly selective and apparently non-selective ion-pair. The remaining nucleobases are used to evaluate whether different bases result in different selectivities. The stereo configuration of the pentamer dT modified with sulfur in each possible position was determined using an assay based on enzymatic digestion using snake venom phosphodiesterase for identifying if the elution order of the different diastereomers depends on the position of the PS modification.

## Materials and methods

### Chemicals

The mobile phase consisted of HPLC-grade acetonitrile from VWR (Radnor, PA, USA) and all water used in this study was deionized water with a conductivity of 18.2 MΩ cm^−1^ from a Milli-Q Advantage A10 water purification system (Merck Millipore, Darmstadt, Germany). Trimethylamine solution (44 wt% in H_2_O, CAS# 75-50-3), triethylamine (≥ 99.5%, CAS# 121-44-8), tripropylamine (≥ 98%, CAS# 102-69-2), tributylamine (≥ 99.5%, CAS# 102-82-9), and acetic acid (99.8–100.5%, CAS# 64-19-7) were all purchased from Sigma-Aldrich (St. Louis, MO, USA). All oligonucleotide samples were purchased from Integrated DNA Technologies (Leuven, Belgium) as “1 μmole DNA Oligo” products delivered in lyophilized and desalted form and used without further purification. Their absolute purity was not established, as it varies with length and composition, but can be expected to be high in the case of a pentamer given a synthesis coupling frequency of > 99%. The general structure of the pentamer used in this study is shown in Electronic Supplementary Material (ESM) Fig. S1.

### Instrumentations

All experiments except the enzyme digestion analysis were performed on an Agilent 1200 Series HPLC System (Agilent Technologies, Palo Alto, CA, USA), configured with a binary pump, a 100-μL injection loop, a diode-array UV detector, and a column thermostat. The temperature of the column thermostat was set to 50 °C and the flow rate to 0.50 mL min^−1^ for all experiments. Analysis of enzymatic digestion samples (section “Stereoselective enzyme digestion”) was performed using a Waters Acquity UPLC H-class system (Waters Corporation, Milford, MA, USA) configured with a quaternary pump, a 10-μL injection loop, a diode-array UV detector, and a column thermostat. The temperature of the column thermostat was set to 50 °C and the flow rate to 0.25 mL min^−1^. A Waters XBridge C_18_ 150 × 2.1-mm, 3.5-μm column was used with the Agilent 1200 system (sections “Tertiary amine screening,” “Retention study using tributylamine with preparative composition analysis,” and “Diastereomer selectivity using triethylamine”). A Waters XBridge C_18_ 100 × 2.1-mm, 5-μm column was used for the enzymatic digestion analysis (section “Stereoselective enzyme digestion”).

## Procedures

### Sample and eluent preparation

All oligonucleotide samples were prepared as stock solutions of approximately 0.7 mg mL^−1^ by dissolving the oligonucleotide in 2 mL of water and vortexing. All samples were kept refrigerated and diluted to 0.1 mg mL^−1^ before injection. A simplified naming convention was used to describe the oligonucleotides. For example, the pentamer of deoxythymidine monophosphate with sulfur in position one and two, 5′-T_S_T_S_TTT-3′, is referred to as T5-1-2S. All eluents were prepared by mass, i.e., by weighing acetonitrile/water and tertiary amine/acetic acid. Molar concentrations of the ion-pair were estimated from the calculated volume of the eluent. Acetonitrile was added first, followed by deionized water. While stirring, acetic acid followed by alkylamine was added. Stirring continued for 1–6 h to allow the different amines to dissolve properly, as verified by ocular inspection. Some eluents containing triethylamine and tributylamine were measured at ${}_w^s\mathrm{pH}$ 7–8, meaning that all pentamer oligonucleotides had a formal charge of − 4, one per phosphate group. ${}_w^s\mathrm{pH}$ is a pH scale in which the electrode is calibrated in water solutions but measured in the eluent containing acetonitrile (MeCN) in this case (see Rosés and Bosch [28] for more discussion of various different pH scales). Eluents were vacuum degassed inline or manually for about 5–10 min before use. The column was equilibrated using at least 100 column volumes when switching between ion-pairs as well as when varying the ion-pair concentration during the design of experiments using TEtAA or TBuAA. Gradient elution mode was used for all experiments to increase robustness as all eluents were prepared fresh daily [29]. In the case of using TEtAA to separate the 16 diastereomers of T5-1-4S (2^4^), gradient elution is necessary to achieve separation.

### Tertiary amine screening

Because TEtAA has previously been shown to promote diastereomer selectivity, it was decided to study a series of tertiary alkylamines. Trimethyl-, triethyl-, tripropyl-, and tributylamine were investigated as ion-pairing reagents. Identical eluents containing 15 mM equimolar amounts of each ion-pair and acetic acid were prepared daily, and a gradient from 5 to 58 v% MeCN at 1% min^−1^ was run for each ion-pair system. Samples T5, T5-2S, A5-2S, C5-2S, and G5-2S were injected separately in one replicate each.

### Stereoselective enzyme digestion

The *R*p and *S*p diastereomers of T5 modified with sulfur at the first, second, third, and fourth phosphate groups were purified by collecting one fraction per peak. Eluent containing 50 mM TEtAA was utilized at a gradient slope of 0.5% min^−1^. Triplicate 100-μL 0.7 mg mL^−1^ injections were made and both peaks were collected after the UV detector. The pooled fractions were analyzed for purity by injecting 5 μL of the undiluted fractions using the same gradient program. Samples were put in the freezer (− 18 °C) until enzymatic digestion experiments started.

Digestion of 3.5-μg oligonucleotide samples was carried out by adding 0.5 U snake venom phosphodiesterase (*Crotalus adamanteus* venom; Affymetrix/USB, Alfa Aesar), 10 U alkaline phosphatase (calf intestinal; New England BioLabs, Ipswich, MA, USA), and MgCl_2_ to a final concentration of 15 mM and Tris-HCl pH 8.8 to a final concentration of 10 mM in a total volume of 50 μL and incubated at 37 °C for 24 h.

Digested samples were analyzed without further sample preparation and were eluted using 50 mM TEtAA during a 25-min program consisting of a 3-min isocratic hold at 3 v% MeCN followed by a linear gradient to 25.5 v% MeCN.

### Retention study using tributylamine with preparative composition analysis

An investigation into the dependence of the gradient slope and concentration of the ion-pair on retention was performed using TBuAA. Chemometrics was applied to be able to quantitatively compare the variation of the retention factor of T5 with various sulfur substitutions. A central composite face-centered experimental design with three center points was used. Gradient slopes of 0.5, 1.5, and 2.5% min^−1^ MeCN were evaluated at 4, 8, and 12 mM concentrations of TBuAA. All eluents were prepared daily. An initial isocratic level of 29 v% MeCN was used to achieve reasonable retention times. Samples of T5, T5-1S, T5-1-2S, T5-1-3S, and T5-1-4S were injected in duplicate. Additional samples of T5, T5-1S, T5-2S, T5-3S, T5-4S, T5-2-3S, A5-2S, C5-2S, and G5-2S were individually analyzed to investigate the effects of the sulfur position and nucleobase type on retention. Uracil was injected as a void volume marker. Peak apex retention times were extracted from each chromatogram and retention factors were calculated. Multiple linear regressions of retention factors were performed using MODDE 11 (Umetrics, Umeå, Sweden) with a 95% confidence level, and non-significant factors were manually removed. The logarithm of the retention factor was described by a second-degree polynomial (see ESM Eq. S2).

To further investigate whether partial diastereomer separation occurred, injections of semi-preparative 100 μL, 0.7 mg mL^−1^ samples containing T5-2S were performed during a 4 mM TBuAA 0.5% min^−1^ MeCN gradient program. Two fractions of the front and tail of the overloaded peak were collected in an auto sampler vial and then reanalyzed using a 50 mM TEtAA 0.5 min^−1^ MeCN gradient. Peak areas were integrated and the relative amounts of *R*p and *S*p diastereomer could be calculated.

### Diastereomer selectivity using triethylamine

To investigate the effect of ion-pair concentration and gradient slope on the retention and selectivity of diastereomer separation, TEtAA was used in the same experimental design as for TBuAA. Gradient slopes of 0.5, 1.5, and 2.5% min^−1^ MeCN were investigated with 20, 50, and 80 mM TEtAA. The initial isocratic amount was 5 v/v% MeCN. Samples of T5, T5-1S, T5-1-2S, T5-1-3S, and T5-1-4S were injected in duplicate. Additional samples of T5, T5-1S, T5-2S, T5-3S, T5-4S, T5-2-3S, A5-2S, C5-2S, and G5-2S were individually analyzed to investigate the effects of the sulfur position and nucleobase type on the diastereomer separation. In all cases in which there were two diastereomers, selectivity could easily be calculated. In all other cases, selectivity was expressed between the first and last detectable peaks in the apparent cluster of peaks. Selectivity was described by the same type polynomial as used to describe the retention above (see ESM Eq. S2).

## Results and discussion

This section is divided into four parts. First, an investigation of how diastereomer selectivity varies with the carbon chain length of tertiary alkylamines is presented, followed by an in-depth investigation of the effect of TBuAA on oligonucleotide retention and selectivity. Third, an investigation of stereo configuration and retention order is presented. Finally, an investigation of TEtAA is presented in which the effects of gradient slope, amine concentration, number of sulfurs, and type of nucleobase on retention and selectivity are investigated.

## Altering diastereomer selectivity by choice of tertiary alkylamine

Oligonucleotides are typically separated using alkyl amines as ion-pairing reagents [10]. Here, the effects of the carbon chain length of the tertiary alkylamine on the retention and diastereomer selectivity were investigated for identical gradients and molar concentrations of the ion-pair.

**Fig. 1:**
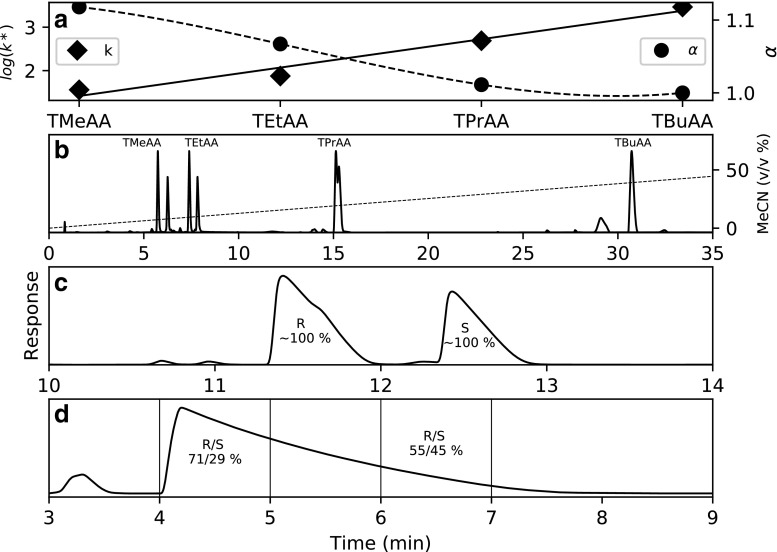
Overview of retention and diastereomer selectivity obtained when separating T5-2S using trimethylammonium acetate (TMAA), triethylammonium acetate (TEtAA), tripropylammonium acetate (TPAA), or tributylammonium acetate (TBuAA): (a) shows how the retention factor of T5 (filled diamonds) and the selectivity (filled circles) of the two diastereomers of T5-2S vary with type of ion pair; (b) shows the chromatogram of injections of T5-2S using different ion-pairing reagents and identical gradients (overlay); (c) shows the overloaded chromatogram of the injection of T5-2S eluted using 50 mM TEtAA and a gradient of 0.5% min^−1^; and (d) shows the same injection as in (c) but instead using 4 mM TBuAA in the eluent; vertical lines indicate the cut-points defining the two fractions

In Fig. 1 (a), the retention factors of T5 are plotted against various tertiary amines carrying different lengths of alkyl chains. The trimethylammonium acetate (TMeAA) ion-pair results in the lowest retention factor and TBuAA the highest (see diamonds in Fig. 1 (a)). The logarithm of the retention factor increases linearly with each methylene group added to the tertiary amine (see inserted linear trend line in Fig. 1 (a)).

To investigate how the carbon chain length of the tertiary amine affects the diastereomer selectivity, we instead studied T5-2S, which has two diastereomers. Regarding the diastereomer selectivity of T5-2S, the trend is reversed: TMeAA has the highest diastereomer selectivity (*α* = 1.12), whereas using TBuAA leads to no measurable selectivity (Fig. 1 (b)). Diastereomer selectivity decreases with increasing carbon chain length of the amine, keeping all other conditions constant.

TEtAA has been shown by several authors to be selective, whereas TBuAA has been shown to be non-selective. The selectivity observed using TMeAA and tripropylammonium acetate (TPrAA) has, to our knowledge, not been reported before.

Based on these results, TMeAA should be the ion-pairing reagent of choice for maximizing the diastereomer selectivity. However, trimethylamine is also the most volatile amine (boiling point 2.9 °C) in this study and will readily evaporate from the eluent containers. This was apparent as TMeAA experiments had the lowest reproducibility between injections. Triethylamine was therefore chosen as a good compromise between robustness, practicality, and acceptable diastereomer selectivity.

The general increase in retention factor with increasing length of the ion-pairing alkylamine carbon chain has previously been quantified for T5 by McKeown [30]. Similar quantitative results have also been for observed for anionic ion-pairing systems [31–33]. Depending on the length, i.e., the hydrophobicity of the alkylamine, the mechanism can be attributed to a combination of ion-pair formation in the mobile phase and a dynamic ion-exchange mechanism as the ion-pairing reagent adsorbs to the stationary phase [31]. Preliminary experiments indicate that triethylamine has weak adsorption (*k* < 1) and tributylamine has moderate (*k* < 4) when eluted in completely unbuffered 10/90 v/v% MeCN/water. More experiments would be required to detail the contribution of each equilibrium to the overall retention factor of the oligonucleotide. The observation that diastereomer selectivity decreases with increasing alkylamine length is more complicated and a plausible explanation will be given in section “Determination of stereo configuration using enzymatic assay.”

## Separation of oligonucleotides using tributylamine

### Varying gradient slope and ion-pair concentration

To investigate the factors controlling retention for the apparent diastereomer-independent ion-pair, an experimental design with varying gradient slopes and ion-pair concentrations was investigated. Gradient slopes of 0.5, 1.5, and 2.5% min^−1^ were investigated at 4, 8, and 12 mM concentrations of TBuAA. Initial experiments were evaluated at 20 mM TBuAA, but tributylamine precipitation was suspected to occur over time. To mitigate this risk, 12 mM TBuAA was used in the design and the eluents were carefully stirred before use.

**Fig. 2:**
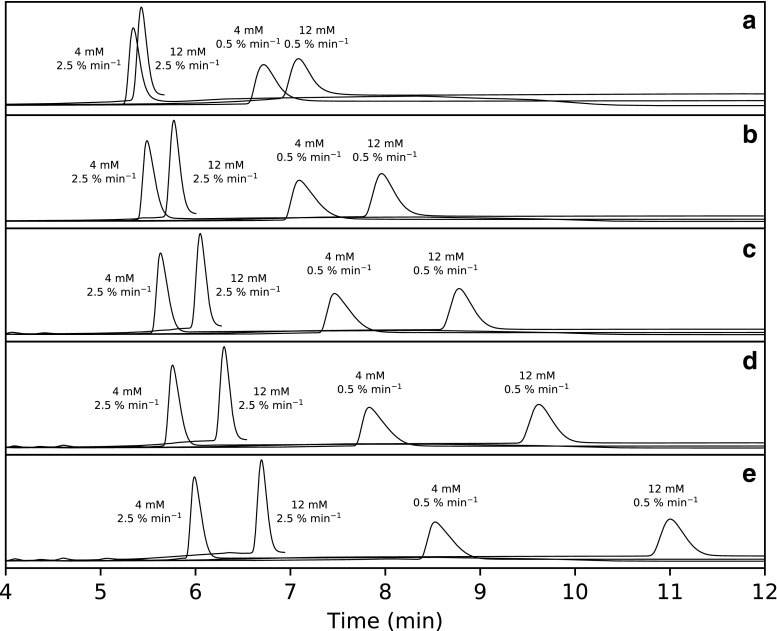
Overview of chromatograms from the experimental design using TBuAA as the ion pair. Low and high concentrations (4 and 12 mM) at high and low gradient slopes (0.5% min^−1^ and 2.5% min^−1^): injection of unmodified oligonucleotide (a) T5, (b) T5-1S, (c) T5-1-2S, and (d) T5-1-3S, as well as (e) completely phosphorothioated oligonucleotide T5-1-4S. The theoretical numbers of peaks in subplots (a)–(e) are 1, 2, 4, 8, and 16, respectively

**Fig. 3:**
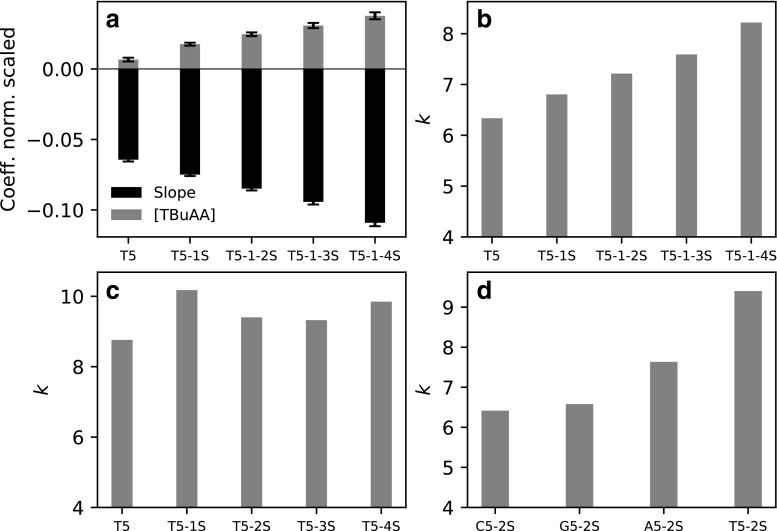
Quantitative results of experiments using TBuAA as the ion pair: (a) shows the model coefficient of gradient slope and TBuAA concentration in Electronic Supplementary Material Eq. S2 for T5 through T5-1-4S; (b) shows the retention factor of T5 modified with 1, 2, 3, and 4 sulfurs obtained at 8 mM 1.5% min^−1^; (c) shows the retention factor for T5 with a single sulfur substitution in different positions obtained at obtained at 12 mM 0.5% min^−1^; and (d) shows the retention factor for the pentamers of A, T, G, and C nucleobases with sulfur substitution at the second phosphate linkage obtained at identical conditions as (c)

Inspecting the chromatogram in Fig. 2 (a), retention of T5 increases with decreasing gradient slope and increases with increasing TBuAA concentration as expected [34]. The center point of 1.5% min^−1^ and 8 mM TBuAA was omitted for clarity but indicates the same trend. The addition of one to four sulfurs increases the retention factor (Figs. 2 (b–e) and 3 (b)). The general increase in retention could probably be attributed to a general increase in hydrophobicity and/or at least two phenomena: (i) weaker hydrogen bonding with water for sulfur than for oxygen [35] and (ii) stronger ion-pairing with alkylamine due to higher charge localization on the sulfur [36]. The wider peak width of T5-2S eluted as a single peak using TBuAA compared to that of individual diastereomer peaks eluted using either TMeAA or TEtAA (Fig. 1 (b)) could be explained by partial diastereomer selectivity. A counterargument is that the peak width of T5 does not increase with increasing number of sulfur substitutions (Fig. 2 (b–e)). Analysis of fractions obtained from the overloaded T5-2S injection (Fig. 1 (d)) shows that the ratio of *R*p and *S*p diastereomers differs between the front and tail of the elution peak. The area ratio of the first- and last-eluting diastereomers is about 71/29 in the front and 55/45 in the rear. If the diastereomers of T5-2S are completely separated using the TEtAA ion-pair, the ratio is 60/40 (see ESM Fig. S3). This evidence suggests that the peak broadening observed when analyzing various pentamers of T5 could be due to partial diastereomer separation. These results demonstrate that the cut-point in a preparative chromatographic method determine the diastereomeric composition of the target component.

Analyzing the retention data obtained in the experimental design reveals that the sensitivity of the retention factor to both TBuAA concentration and gradient slope increases with increasing number of sulfurs (Fig. 3 (a)).

### Position of sulfur and type of nucleotide

We wanted to evaluate whether the position of a single sulfur substitution and the type of nucleobase affect retention when using TBuAA. The results indicate that the retention factor is at its maximum if the sulfur is substituted at either the 5′ or 3′ end of the oligonucleotide, versus being substituted in the center of the oligonucleotide at position 2 or 3 (Fig. 3 (c)). A possible explanation for this observation is that the anionic sulfur is less shielded, i.e., more likely to interact with the ion-pairing reagent, if it is substituted at either end versus in the center of the oligonucleotide. Regarding the type of nucleobase, the retention factor was found to increase for cytosine < guanine < adenine < thymidine (Fig. 3 (d)). This pattern is similar to what was observed by Gilar et al. for unmodified oligonucleotides [37].

## Determination of stereo configuration using enzymatic assay

In investigating how the stereo configuration (*R*p, *S*p) relates to the retention order of the diastereomers, it was decided to limit the investigation to single-sulfur–modified oligonucleotides, as the complexity of the analysis increases dramatically with each additional added sulfur. However, the same investigation was performed for sulfur substitutions at all four possible positions (i.e., T5-1S, T5-2S, T5-3S, and T5-4S). A semi-preparative setup was devised to collect fractions using the 150 × 2.1-mm column. Using a shallow gradient of 0.5% min^−1^ and a TEtAA concentration of 50 mM, 100 μL of 0.7 mg mL^−1^ of each oligonucleotide was injected. The chromatogram for T5-2S indicates that with this injected amount and under these conditions, the peaks are Langmuirian and that the conditions should allow for high yield and purity (see Fig. 1 (c)). In the case of T5–2S (Fig. 1 (c)), the first-eluting peak was collected between 11.5 and 12.0 min. and the second peak between 12.5 and 13.0 min. This was repeated twice until about 750 μL of each fraction had been collected. This procedure was repeated for T5-1S, T5-3S, and T5-4S with appropriate collection times (see ESM Fig. S4).

The stereo configuration of each fraction was determined by enzymatic digestion with snake venom phosphodiesterase and alkaline phosphatase. Snake venom phosphodiesterase is a 3′ to 5′ exonuclease, phosphorothioate modified oligonucleotides are cleaved with lower efficiency. Stereo configuration of the phosphorothioate group can be determined due to more efficient digestion of the *R*p compared to the *S*p configuration [38]. Snake venom phosphodiesterase from *Crotalus adamanteus* can also act as an endonuclease on single-stranded oligonucleotides [39, 40]. Digestions were performed at high enzyme concentration and long reaction times in order to completely digest unmodified oligonucleotides as well as modified oligonucleotides in *R*p configuration. Alkaline phosphatase was added to remove residual unmodified and modified phosphate groups at the 5′ end of the digested fragments [41]. In this way, only nucleosides were expected to be found after digestion of phosphorothioated oligonucleotides in *R*p configuration while the *S*p diastereomer was expected to produce the dinucleotide TsT in addition to the nucleosides. Digestion of the first-eluting peak of T5-1S (fraction 1) resulted in a single peak at 2.2 min, containing the nucleoside deoxythymidine (see ESM Fig. S5b). Enzymatic treatment of the second-eluting peak (fraction 2) resulted in two peaks, the first overlapping the deoxythymidine peak from fraction 1 and the second eluting at 8.8 min (see ESM Fig. S5b). The second peak was attributed to the T_S_T. Digestion of all other samples gave the same digestion pattern (data not shown). This shows that the *R*p diastereomer always elutes first, verifying and complementing the observations initially made by Stec et al. [15].

Several plausible explanations to the observation that diastereomer selectivity decreases with increasing alkylamine length could be proposed. Many observations support an explanation that hydrophobic interactions dominate. The first is that (i) separation using shorter alkyl amines results in less ionic interactions due to lower adsorption of ion-pair on the stationary phase and that (ii) lower amount ion-pair is formed in the of mobile phase [42]. When using longer alkyl amines, the hydrophobic contribution to retention decreases and stronger ion-exchange like contributions increase which then suppresses diastereomer separation. Extrapolating the selectivity trend in Fig. 1a to ammonium further supports the hydrophobic model. Ammonium would provide very weak ion-pairing and elution would be performed at very low acetonitrile amounts.

However, the observation that diastereomers of short PS-modified oligonucleotides also can be separated using silica based DEAE ion-exchange chromatography [16, 22] as well as partially by capillary electrophoresis [23] contradicts the hydrophobicity explanation model. Kanehara et al. [16] found that the *S*p diastereomer of a single sulfur modified pentamer had longer retention time than *R*p, just as has been consistently observed in this and previous studies [16, 17, 19–21].

Without detailed knowledge about each possible adsorption equilibrium, it is not possible to determine the exact mechanisms behind diastereomer selectivity. Additional experiments would be necessary.

Also, structural studies of PS-modified oligonucleotides could give some indication of why the *S*p diastereomer has a longer retention time than does the *R*p diastereomer if they are separated exclusively based on their charge. Using NMR, Lan et al. [43] investigated a 10-nucleotide-long double-stranded DNA heteromer with a single PS modification in the sense and complementary antisense strands. They found that when both strands were in *R*p configuration, the sulfur of the phosphorothioate group is positioned on the inside of the dsDNA helix, whereas in the *S*p configuration, the group is positioned facing the outside. Given that the favored resonance structure of the phosphorothioate groups is O=P–S^−^ [36], ion-pairing interaction will likely be stronger with the *S*p than the *R*p diastereomer. This hypothesis could explain the observed retention order. However, exposing the more hydrophobic sulfur in *S*p diastereomer to the solution will enhance the hydrophobic interaction compared to *R*p diastereomer where the more hydrophilic oxygen is exposed. In addition, it remains to be investigated whether Lan et al.’s structural results can be extrapolated to short single-stranded oligonucleotides, as only a few studies have examined the secondary structure of single-stranded oligonucleotides.

### Separation of diastereomers using triethylamine

#### Degree of sulfur substitution

For understanding how the degree of sulfur substitution affects the selectivity, T5 was substituted with an increasing number of sulfurs from the 5′ to 3′ end of T5. As a reference, the retention of unmodified T5 was studied in an experimental design using 20, 50, and 80 mM TEtAA at 0.5, 1.5, and 2.5% MeCN min^−1^. Significant model coefficients are presented in Fig. 5.

**Fig. 4:**
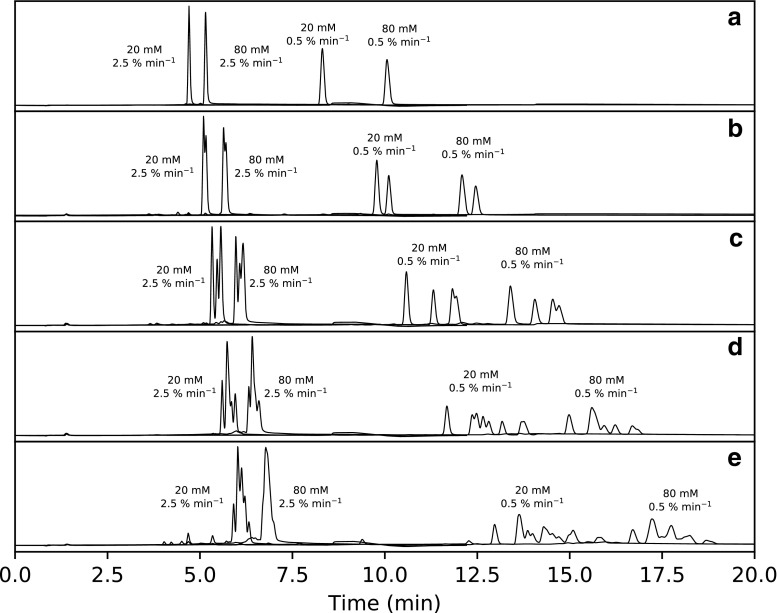
Summary of chromatograms for the low and high ion-pair concentrations (i.e., 20 and 80 mM) at low (0.5% min^−1^) and high gradient slopes (2.5% min^−1^). Chromatograms of unmodified oligonucleotide (a) T5, (b) T5-1S, (c) T5-1-2S, and (d) T5-1-3S, as well as (e) completely modified oligonucleotide T5-1-4S. The theoretical numbers of peaks in subplots (a)–(e) are 1, 2, 4, 8, and 16, respectively

Before discussing the experimental design, let us consider some chromatograms used to obtain retention and selectivity data for the model (see Fig. 4). Figure 4 (a) shows chromatograms from injections of unmodified T5 and Fig. 4 (b–e) injections of modified T5 with an increasing number of sulfurs from the 5′ to 3′ end. The first observation is that the retention time of peaks or clusters of peaks increases with an increasing number of sulfurs, similar to what was observed for TBuAA. Studying the simplest case, T5-1S (Fig. 4 (b)), it is apparent that diastereomer selectivity increases with decreasing gradient slope but varies only slightly with TEtAA concentration. The center point of 50 mM TEtAA was omitted for clarity but indicates the same trend (see ESM Fig. S6). The addition of a second sulfur (T5-1-2S) should give four diastereomers, and these four peaks are only observed at the 0.5% min^−1^ gradient (Fig. 4 (c)). At the steeper gradient, i.e., 2.5% min^−1^, only three peaks are visible due to partial or complete co-elution of the third and fourth peaks. Adding a third sulfur gives eight diastereomers. These are also only observed for the shallow gradient but now only with 20 mM TEtAA (Fig. 4 (d)), indicating that diastereomer selectivity decreases with the increasing concentration of TEtAA. Increasing the TEtAA concentration and/or increasing the gradient slope will result in a loss of diastereomer selectivity up to a point at which there is partial resolution and the convolution of just three peaks. When analyzing the fully phosphorothioated oligonucleotide, 16 diastereomers are expected but at most eleven are observed with a slope of 0.5% min^−1^ and 20 mM TEtAA (Fig. 4 (e)). Increasing the slope to 2.5% min^−1^ with 80 mM TEtAA in the eluent results in almost full co-elution into one peak. Another observation is that the retention of the cluster of peaks increases with an increasing number of sulfurs and increasing amount of TEtAA in the eluent.

**Fig. 5:**
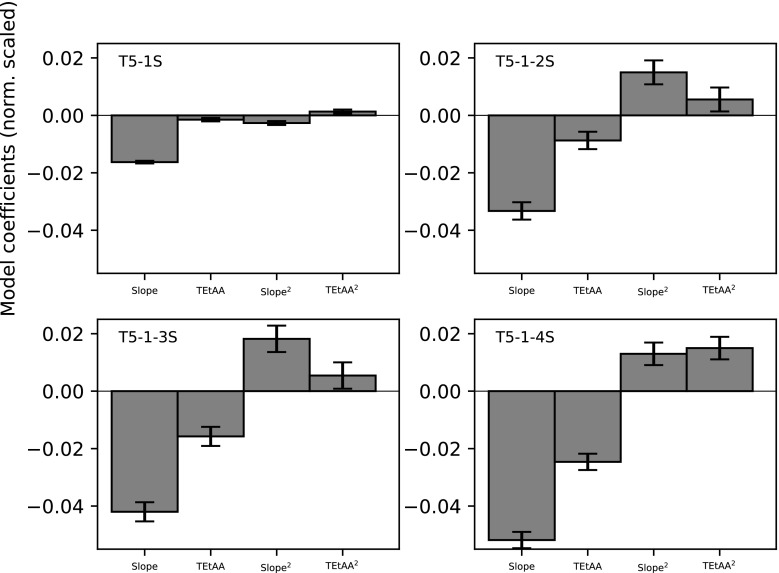
The normalized and scaled coefficients from Electronic Supplementary Material Eq. S2 are presented for diastereomer selectivity from T5-1S through T5-1-4S. For T5-1S, selectivity was calculated between the two diastereomers, and for all others as the selectivity between the last- and first-eluting peaks

For the application of ESM Eq. S2, the selectivity values used as inputs were calculated in different ways. For T5-1S (Fig. 4 (b)), selectivity is a direct measure of the selectivity between the *R*p and *S*p diastereomers, but for all the others, selectivity is measured between the first- and last-eluting peaks of the cluster of peaks and hence is a measure of the total peak broadening due to diastereomer separation. Evaluating the coefficients of Eq. S2 summarized in Fig. 5, the gradient slope is the most important factor followed by the amount of TEtAA in the eluent. Both these factors decrease the selectivity, with the effect increasing with each addition of sulfur to T5. All models also suggest an increase in selectivity due to the quadratic terms of the gradient slope and TEtAA concentration. The impact of the quadratic terms is also evident from the curvature of the contour plots of selectivity (see ESM Fig. S7).

### Position of sulfur and type of nucleotide

**Fig. 6:**
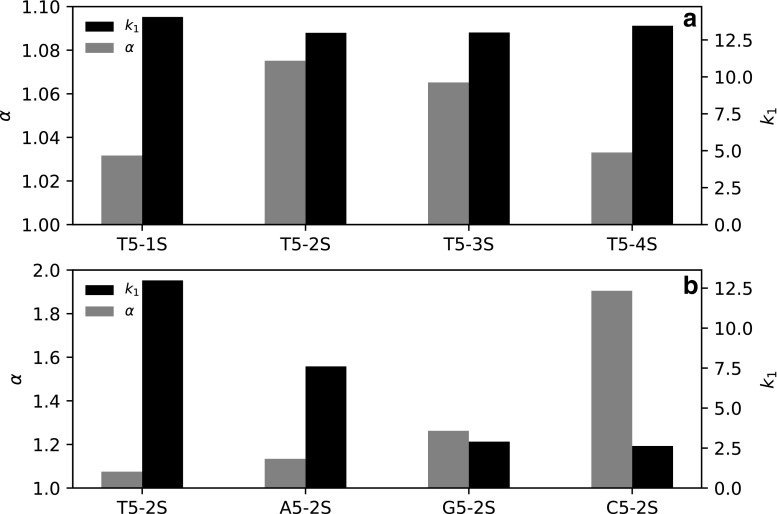
Overview of how selectivity varies with the sulfur position and nucleobase type when eluting the pentamer using TEtAA: (a) shows the retention factor of the first-eluting peak (black) and selectivity (gray) for all four possible sulfur positions in T5; (b) shows the retention factor of the first-eluting peak (black) and selectivity (gray) for pentamers of thymidine, adenine, guanidine, and cytosine with sulfur substitution at the second phosphate linkage

It was also relevant to investigate how the position of sulfur substitution affected the selectivity. Single injections of T5 with sulfur at one of positions 1 to 4 were performed at 0.5% min^−1^ and 80 mM TEtAA. The results indicate that the retention factor of the first-eluting peak (*R*p) is larger if the sulfur is substituted at either the 5′ or 3′ end of the oligonucleotide, versus being substituted in the center of the oligonucleotide at position 2 or 3 (Fig. 6 (a)). The opposite trend is observed for diastereomer selectivity, with the highest selectivity being observed when sulfur is substituted in position 2, closely followed by position 3. Substitution at either the 5′ or 3′ end gives the lowest selectivity. A plausible explanation is that the anionic sulfur is more exposed, regardless of the *S*p or *R*p configuration, when it is substituted at the ends rather than the center of the oligonucleotide. Regarding the type of nucleotide, the retention order for the first-eluting peak was found to be thymidine > adenosine > guanine > cytosine; regarding diastereomer selectivity, the order was the opposite (Fig. 6 (b)). The relative abundances of the *R*p and *S*p diastereomers were found to be about 59:41 of the first- and last-eluting peaks by integrating and averaging all chromatograms obtained for T5-1S through T5-4S. Approximately the same ratio was also found when integrating either A5-2S, C5-2S, or G5-2S (ESM Figs. S3 and S8). This ratio confirms the earlier work of Wilk et al., who found similar ratios for tri- and tetramers of PS-modified oligonucleotides [21]. Quantification of diastereomer distribution could be a relevant approach to evaluate the stereospecificity of the synthesis, especially given that diastereomers could have different physicochemical and pharmacological characteristics [11–14].

## Conclusions

A fundamental investigation of factors influencing the ion-pair RPLC separation of diastereomers of pentameric oligonucleotides as model substances was presented. The properties of the type and concentration of ion-pair reagent in the eluent and the properties of the oligonucleotide model solutes were in focus. Four different tertiary alkylamines in their ammonium acetate form was investigated. Trimethylamine gave the lowest retention and highest diastereomer selectivity, while tributylamine gave no apparent selectivity. However, as also demonstrated in this study, we could show even tributylamine has partial selectivity.

A design of experiments was carried out using tributylamine to evaluate whether any conditions promote diastereomer selectivity. No obvious conditions were found, but retention was shown to increase with the number of phosphorothioate substitutions. Interestingly, the position of the sulfur substitution was found to change the retention. Substitution at the first or fourth phosphate linkage gave higher retention than did substitution at position two or three.

Finally, an experimental design treating gradient slope and ion-pair concentration was performed using triethylamine. Analysis showed that diastereomer selectivity increases with both decreasing gradient slope and decreasing ion-pair concentration. This conclusion is valid when modifying the pentamer with 1, 2, 3, or 4 sulfurs at the phosphate linkage. If substituting single oxygen for sulfur, we found that selectivity was higher if the substitution was made at the second or third phosphate linkage than if it was made at the ends of the pentamer that is at the first or fourth phosphate linkage. The retention order of the two diastereomeres was, however, not effected by the sulfur position and was consistently found to be *R*p followed by *S*p. The type of nucleobase also affected the selectivity of single phosphorothioated pentamers. A homomer of cytosine had almost twice the selectivity as did a homomer of thymine. Cytosine and guanine monomers have almost identical retentions, but cytosine has a 60% higher diastereomer selectivity. This observation indicates that peak broadening due to partial diastereomer separation will be dependent on the sequence of the oligonucleotide analyzed. For example, a cytosine rich heteromer could be expected to show larger peak broadening than a thymine rich.

In this study, we have systematically shown how to both increase and decrease diastereomer separation by both the choice of ion-pair and method conditions. We believe that the present observations could provide an important foundation for understanding and further study the underlying separation mechanisms.

Another important implication of this work is for the preparative separation of phosphorothioated oligonucleotides. This topic is an ongoing project in our research group.

## Electronic supplementary material

ESM 1 (PDF 1371 kb)
