# Supplementary material for: Investigation of factors influencing the separation of diastereomers of phosphorothioated oligonucleotides
Source: Anal Bioanal Chem. 2019 Apr 24;411(15):3383–94. doi: 10.1007/s00216-019-01813-2 (PMC6543027; doi:10.1007/s00216-019-01813-2)
Supplement: Supplementary file 1 — (PDF 1371 kb) [file 216_2019_1813_MOESM1_ESM.pdf]

Analytical and Bioanalytical Chemistry

Electronic Supplementary Material

Investigation of factors influencing the separation of diastereomers of phosphorothioated oligonucleotides

Martin Enmark, Maria Rova, Jörgen Samuelsson, Eivor Örnskov, Fritz Schweikart, Torgny Fornstedt

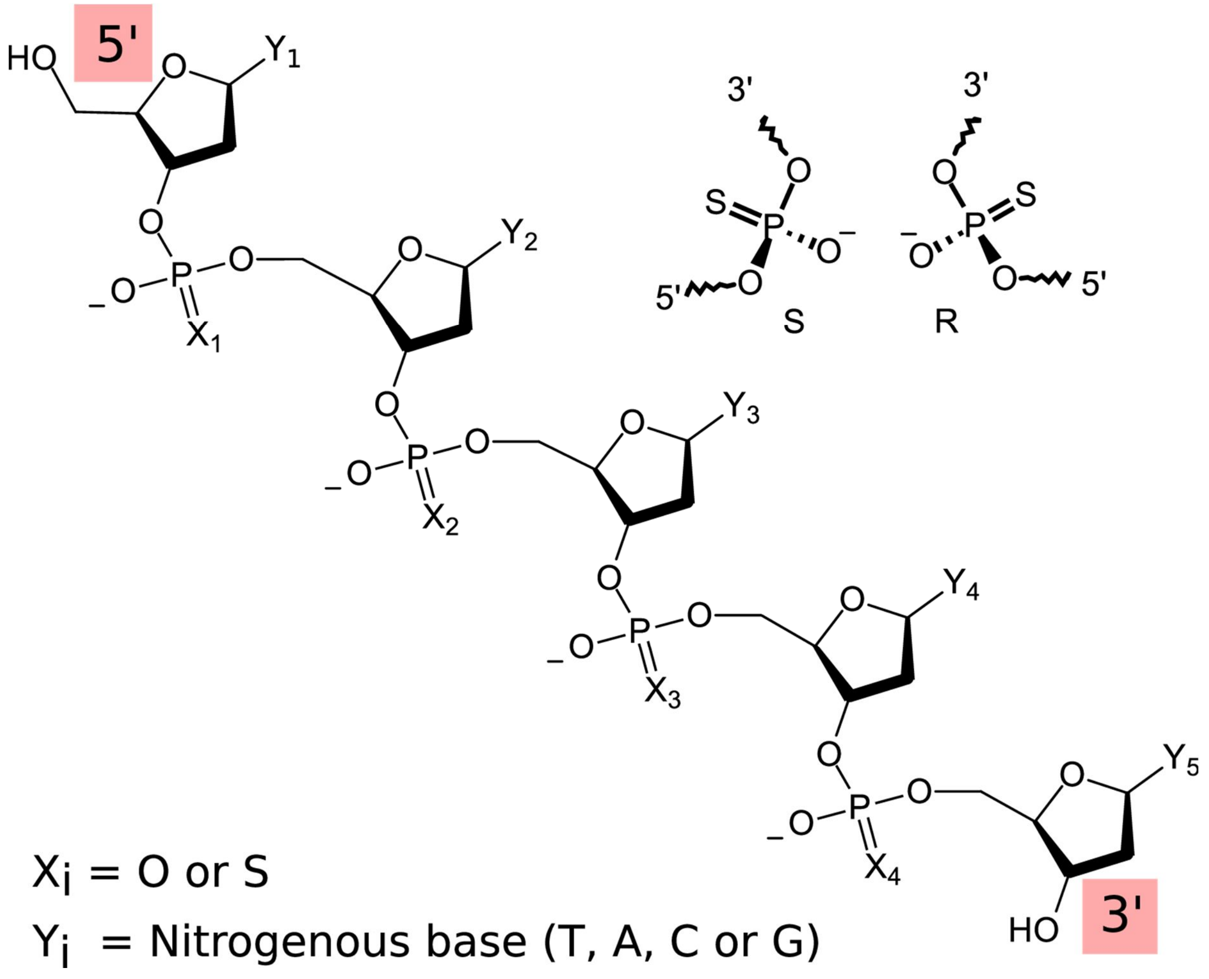

Fig. S1 General structural formula of the oligonucleotides investigated in this study

Equation S2

The logarithm of the retention factor or selectivity (non-transformed) was described by the following second-degree polynomial used in the Experimental Design

$$Response = \alpha_1 S + \alpha_2 C_{IPR} + \alpha_3 S^2 + \alpha_4 C_{IPR}^2 + \alpha_5 S C_{IPR} + \beta \qquad (1)$$

where S is the gradient slope of MeCN (v/v% min^{-1}) and C_{IPR} is the concentration of the ion-pairing reagent in mM. Model coefficient α and model constant β.

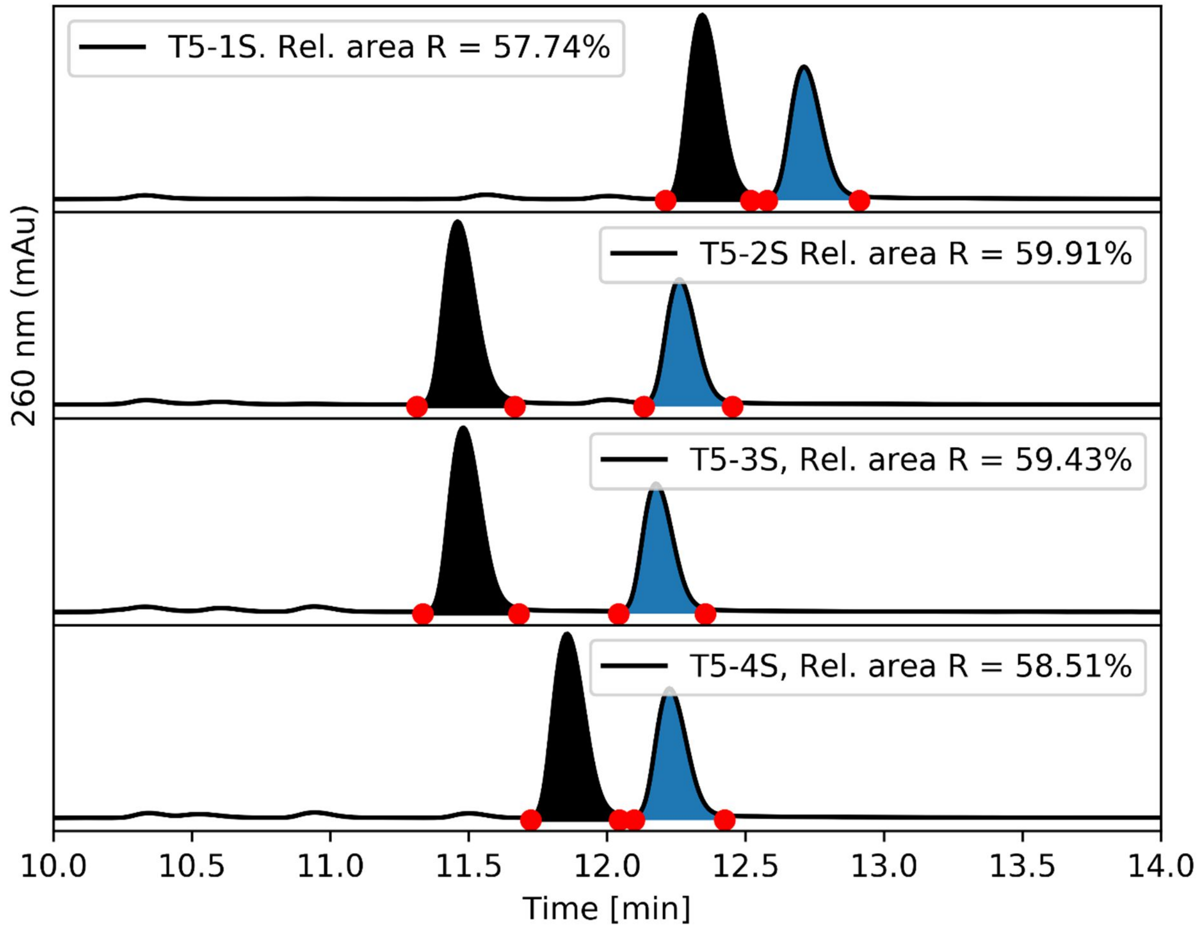

Fig. S3 Integrated peaks of *R*p and *S*p diastereomers from T5-1S through T5-4S showing the relative area of the first-eluting *R*p peak; analysis using an 80-mM triethylammonium acetate (TEtAA) 0.5% min^{-1} gradient

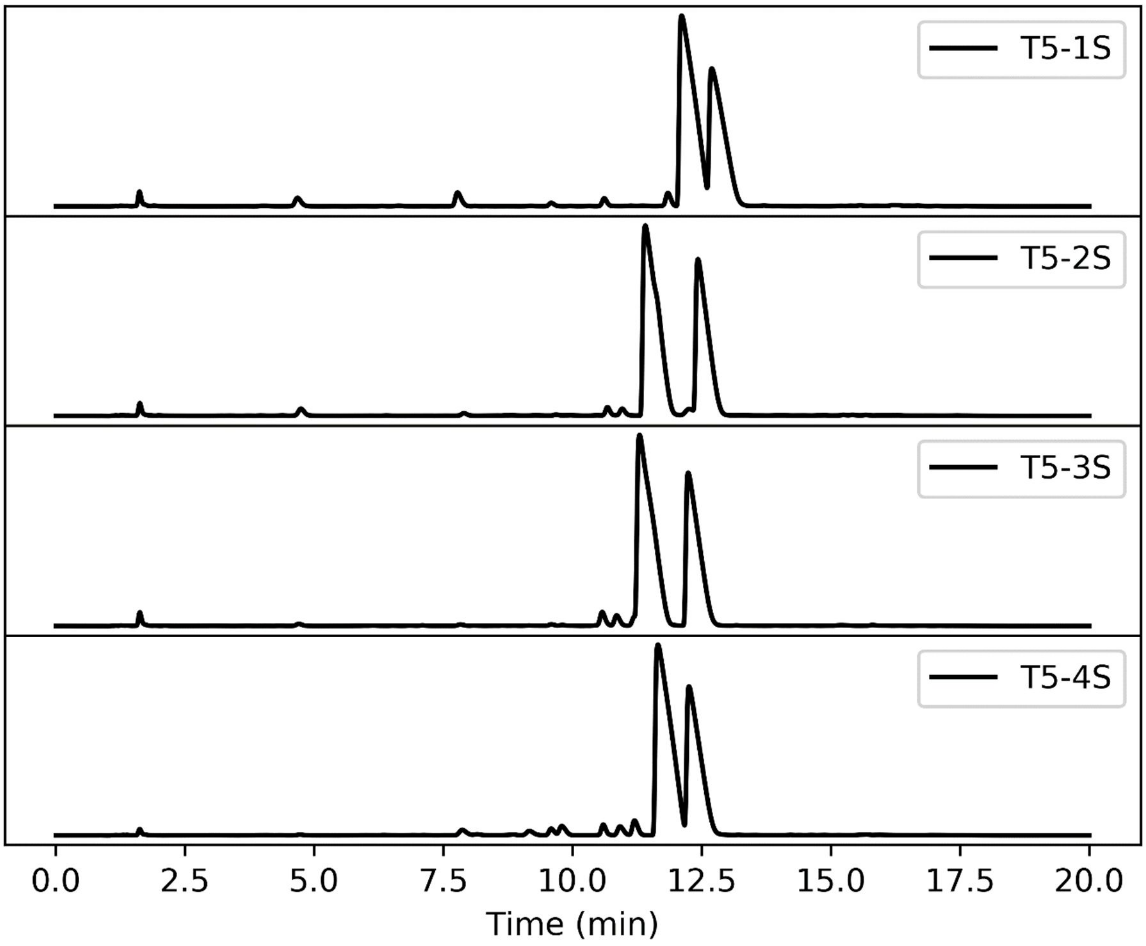

Fig. S4 Chromatograms of column-overloaded injections of T5-1S, T5-2S, T5-3S, and T5-4S, each fractionated for SVPDE analysis; preparative injections of 100 µL of 0.7 mg mL^{-1} eluted in a 50-mM TEtAA 0.5% min^{-1} gradient

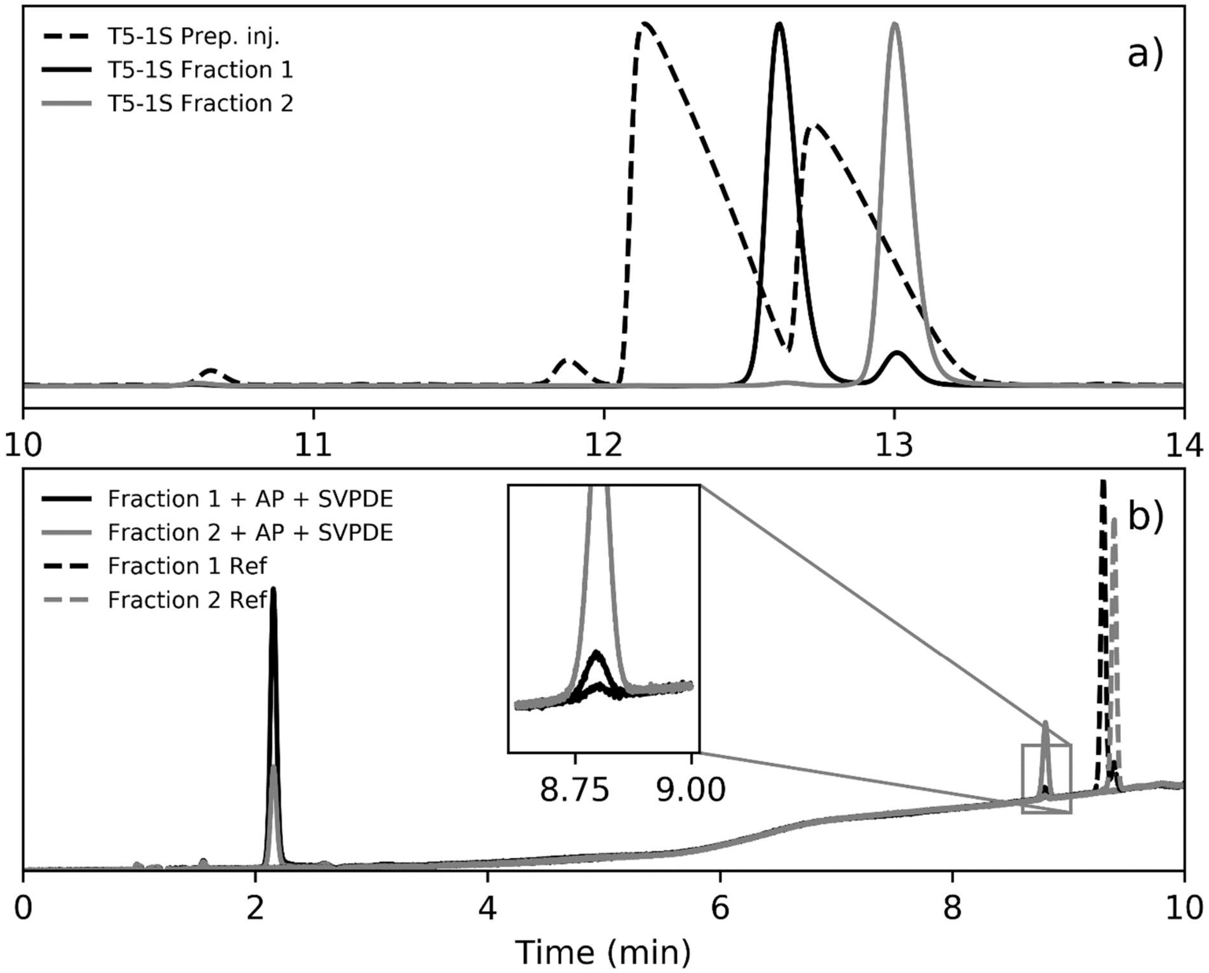

Fig. S5 Results of the enzymatic digestion of the two diastereomers of T5-1S. Subplot (a) shows an overlay of the semi-preparative chromatogram of T5-1S with the re-injected fractions collected from the first and second peaks (normalized response). Both experiments were performed using 50-mM TEtAA and a 0.5% min^{-1} gradient (column: XBridge C_{18} 150 × 2.1 mm, 3.5 µm). Subplot (b) shows an analysis of the two fractions treated with alkaline phosphatase (AP) and snake venom phosphodiesterase (SVPDE) using 50-mM TEtAA during a 25-min program consisting of a 3-min isocratic hold at 3 v% MeCN followed by a linear gradient to 25.5 v% MeCN (column: XBridge C_{18} 100 × 2.1 mm, 5 µm)

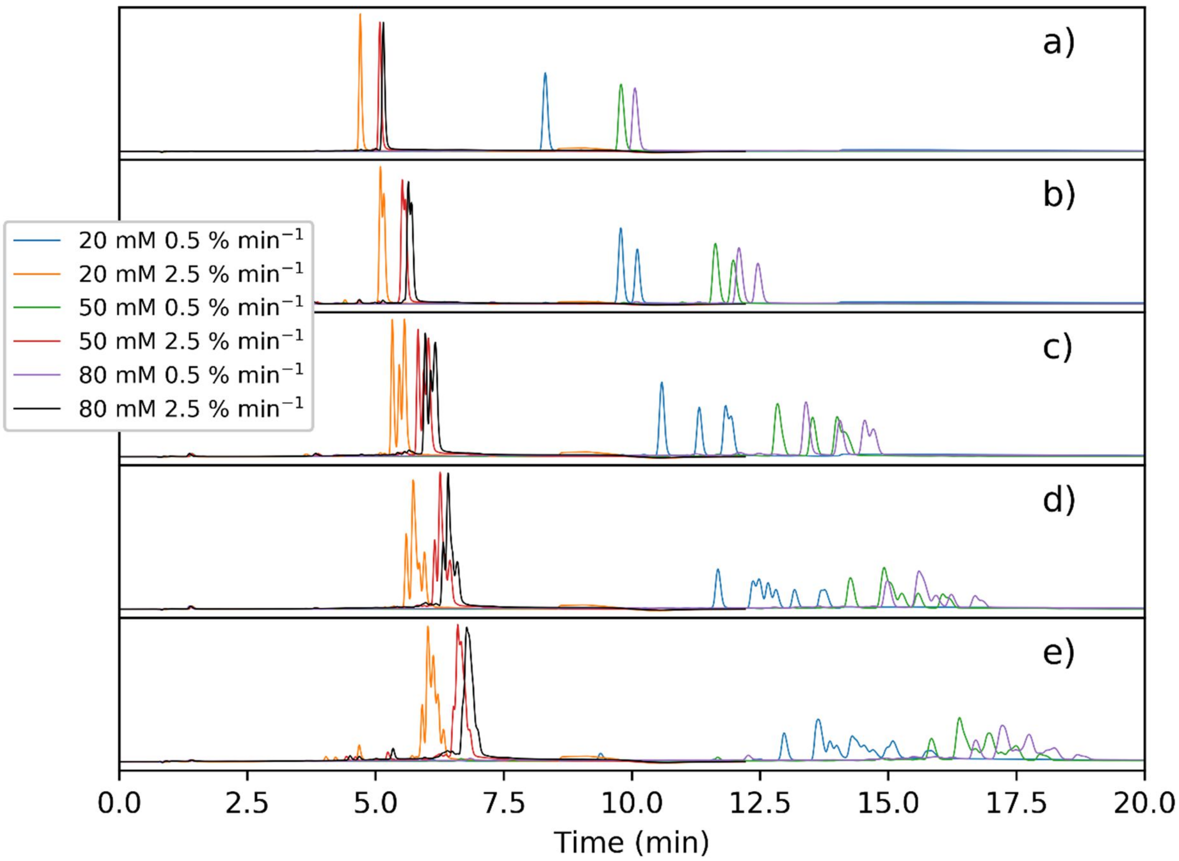

Fig. S6 Summary of chromatograms for the low, medium, and high ion-pair concentrations (i.e., 20, 50, and 80 mM) at low (0.5% min^{-1}) and high gradient slopes (2.5% min^{-1}), with each peak or cluster of peaks labelled accordingly. Injection of completely unmodified oligonucleotide (a) T5, (b) T5-1S, (c) T5-1-2S, and (d) T5-1-3S, as well as (e) completely modified oligonucleotide T5-1-4S; theoretical numbers of peaks (a)–(e) are 1, 2, 4, 8, and 16, respectively

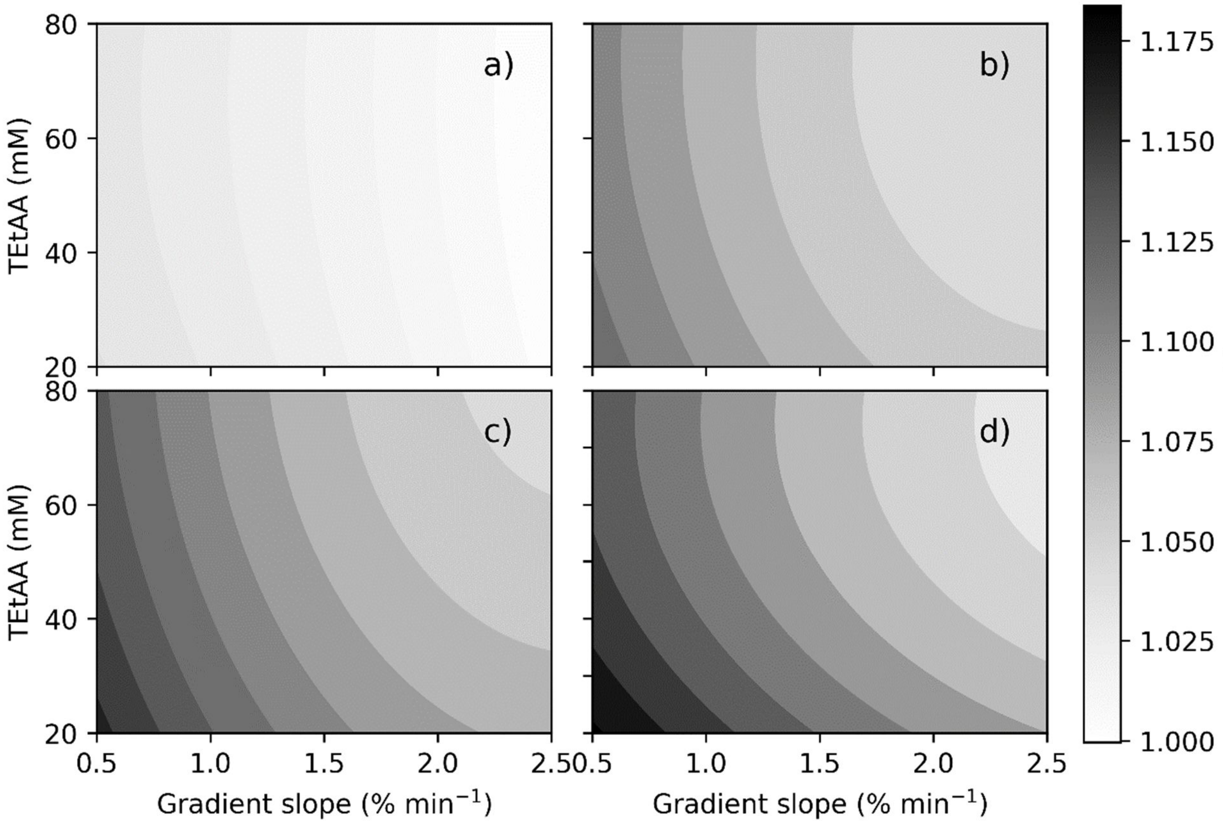

Fig. S7 Contour surfaces showing the selectivity between the first- and last-eluting peaks for T5 modified to vary with gradient slope and TEAA concentration. Interpolation of experimental data using multiple linear regression (see Equation S2). Plots (a)–(d): T5-1S, T5-1-2S, T5-1-3S, and T5-1-4S, respectively

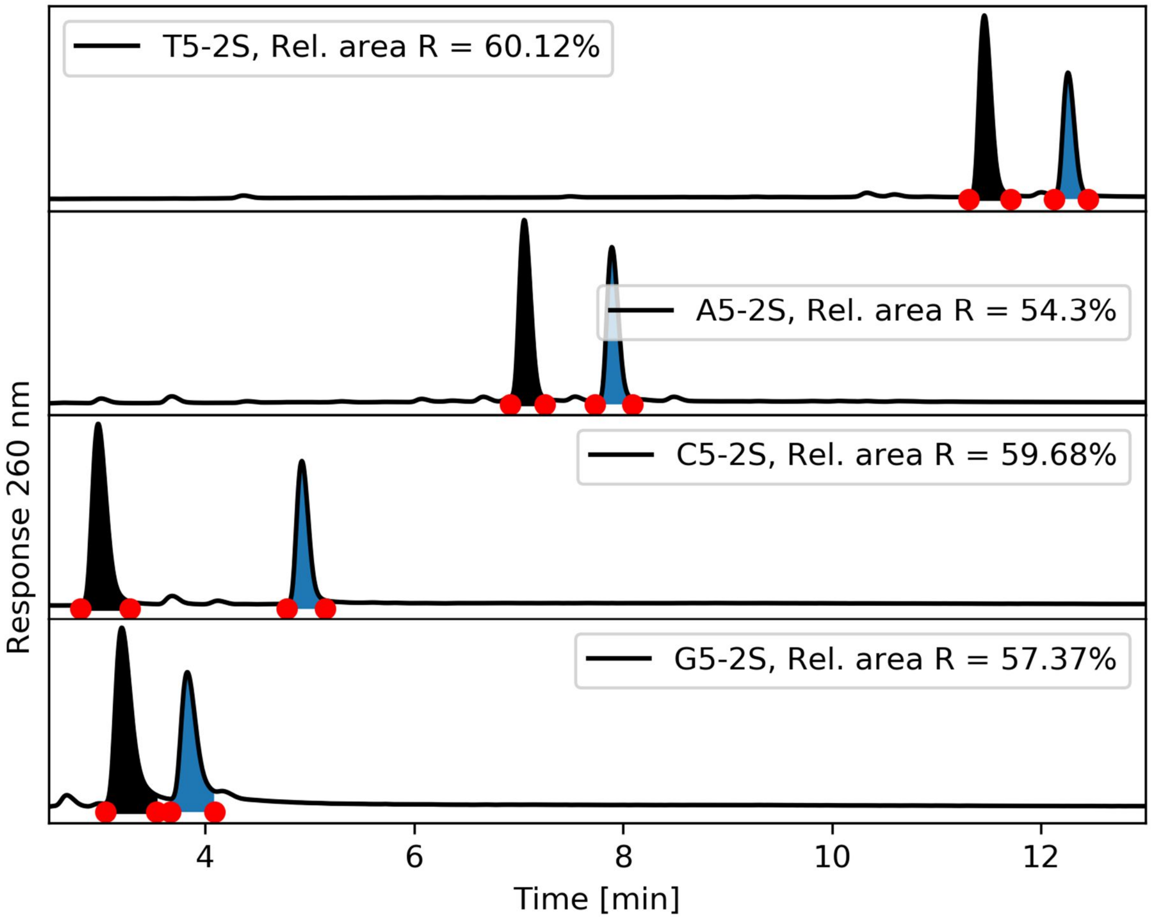

Fig. S8 Integrated chromatograms of T5-2S, A5-2S, C5-2S, and G5-2S showing the relative area of the first-eluting peak; analysis using an 80-mM TEtAA 0.5% min^{-1} gradient
